# Stability of adenine-based cytokinins in aqueous solution

**DOI:** 10.1007/s11627-015-9734-5

**Published:** 2016-02-04

**Authors:** David S. Hart, Andrew Keightley, Daryl Sappington, Phuong T. M. Nguyen, Charleen Chritton, Gary R. Seckinger, Kenneth C. Torres

**Affiliations:** PhytoTechnology Laboratories, 9245 Flint Street, Overland Park, KS 66214 USA; Biological Mass Spectrometry and Proteomics Facility, University of Missouri at Kansas City, Kansas, MO 64110 USA

**Keywords:** Cytokinin, Stability, HPLC, FTIR, Mass spectrometry

## Abstract

Since the isolation of the first cytokinin almost 60 yr ago, cytokinins have become critically important for ornamental and agricultural crops in plant tissue culture. Despite the extensive research on this class of compounds, little information is available on the chemical stability of cytokinins in solution or following an autoclave cycle with Murashige and Skoog (MS) basal medium. This work describes the stability in aqueous solutions of five widely used adenine-based cytokinins: trans-zeatin (tZ), 6-(γ,γ-dimethylallylamino) purine (2iP), kinetin, benzyladenine (BA), and *m*-topolin. High pressure liquid chromatography (HPLC) and electrospray ionization-mass spectrometry (ESI-MS) were used to quantify and identify their degradation. BA, kinetin, 2iP, and *m*-topolin were stable at 1.0 mg mL^−1^ in 0.05 N KOH, with no statistically significant concentration changes (*p* > 0.05) after 90 d of storage at temperatures of −20°C, 2−6°C, or 25°C. The cytokinin tZ was used as a model compound to evaluate stability under alkaline and acid conditions as well as after repeated freeze-thaw cycles. Trans-zeatin retained >90% of the initial concentration of 1.0 mg mL^−1^ when dissolved in 0.01 N KOH and stored at −20°C and 2–6°C for 90 d, with only the 2–6°C temperature treatment showing a statistical significant concentration change (*p* = 0.03). The 1.0 mg mL^−1^ tZ solution in 0.01 N KOH was stable through six repeated freeze-thaw cycles over 90 d without any significant change in concentration compared to the initial freeze-thaw. Yet, tZ showed highly significant concentration changes when dissolved at 50 mg mL^−1^ and 0.5 N KOH. All of these adenine-based cytokinins showed exceptional stability following an autoclave cycle at 121°C, 110 kPa for 30 min when in solutions of 1.0 mg mL^−1^ in 0.05 N KOH, with no significant degradation detected. Trans-zeatin was also found to be stable after one autoclave cycle with 1× MS-basal salts.

**Electronic supplementary material:**

The online version of this article (doi:10.1007/s11627-015-9734-5) contains supplementary material, which is available to authorized users.

## Introduction

Cytokinins are a class of plant-growth regulators that were discovered because of their ability to enhance cell division in plant-tissue culture (Miller *et al.*^1955^). Since the discovery of cytokinins, their disruption of apical dominance (Wickson and Thimann 1958), their biosynthesis from tRNA (Skoog and Armstrong 1970; Letham and Palni 1983) and *de novo* (Takei *et al.*^2001^; Takei *et al.*^2004^), as well as their role in plant development (Werner *et al.*^2003^; Besnard *et al.*^2014^) and signal transduction (Kakimoto 1996; Brandstatter and Kieber 1998; Hwang and Sheen 2001) have become well-defined processes. The use of cytokinins for the maintenance of the shoot apical meristem (Shani *et al.*^2006^), and their *in vivo* metabolism (Mok and Mok 2001) in general have also become better understood. In spite of our knowledge of these processes at the cellular level, little is known about the chemical stability of cytokinins in solution or their physical stability during storage or an autoclave cycle. Though some phenylurea derivatives (e.g., thidiazuron, 4-CPPU) display cytokinin activity (Mok *et al.*^2005^), they do not occur naturally, whereas some adenine-based cytokinins do occur naturally. Adenine-based cytokinins also represent the majority of cytokinins commercially sold.

Cytokinins promote cell division, shoot organogenesis, and shoot proliferation over time. The resultant reduction in production scale-up time is why cytokinins have become so essential in the commercial plant tissue culture industry. Cytokinins are also very potent. They are typically used at hundreds of nmoles l^−1^ to tens of μmoles l^−1^ in tissue culture and endogenous levels in plants exist generally from 1–1000 pmol gDW^−1^ (Shukla and Sawhney 1992; Arthur *et al.*^2007^). It is well-established that, when combined with auxins, very low cytokinin levels can induce a plant tissue to adopt a range of morphogenic states (Skoog and Miller 1957). Since cytokinins are exceptional mediators of these effects, the low concentrations used in culture can possibly be affected by different preparations of concentrated stock solutions and different storage conditions, potentially shifting morphogenic outcomes and causing variable results.

Plant tissue culture has been affected by irreproducible protocols and aberrant growth since its inception (Gautheret 1983; Thorpe 2006; Bairu and Kane 2011). Though somaclonal variation, a consequence of genetic and epigenetic changes (Larkin and Scowcroft 1981; Kaeppler and Phillips 1993; Tanurdzic *et al.*^2008^; Smulders and De Klerk 2011) has been implicated in growth variability, cytokinins have also been associated with aberrant growth such as shoot-tip necrosis (Bairu *et al.*^2011^) and inhibition of root development in culture (Bollmark and Elisasson 1986). Given that cytokinin solutions are prepared using inconsistent protocols and storage conditions that vary from laboratory to laboratory it is possible that cytokinin instability could be responsible for some variability in plant tissue culture responses. In some cases, cytokinin stability may have a significant impact on the reproducibility of tissue culture protocols as well as commercial production for food crops. Therefore it is important to address the stability profiles of these compounds in solution for dependable use in plant tissue culture research as well as commercial production.

There has been a substantial amount of research studying the metabolic degradation of cytokinins. For reviews see Mok and Mok (2001), Auer (2002), and Sakakibara (2006), and Frébort *et al.* (2011). However, it is difficult to ascertain or quantify the degradation of adenine-based cytokinins in solution through biological assays because adenine, the main breakdown product, is known to have shoot proliferation and anti-auxin activities (Murashige 1974). Therefore it is useful to characterize the chemical stability of cytokinins in solutions commonly used in plant tissue culture using non-biological analytical techniques.

There have been reports on the degradation of adenine-based cytokinins in aqueous solution caused by oxidation of tZ with KMnO_4_ (van Staden *et al.*^1982^) and peroxidases (van Staden and Forsyth 1985). Yet degradation studies to evaluate cytokinin stability in typical storage conditions have not been published. Furthermore, the stability during autoclaving of some of these cytokinins has been studied (kinetin in Miller *et al.*^1955^; zeatin riboside in Miller 1974), but most references in the literature are anecdotal with respect to their being (tZ and 2iP in Iliev *et al.*^2010^) or not being (BA and kinetin in Panaia *et al.*^2011^) heat-labile. Because of the limited information available on the chemical stability of cytokinins in solution or their physical stability following an autoclave cycle, the present work was undertaken to determine the stability of five widely used adenine-based cytokinins in aqueous solutions: trans-zeatin (tZ), 6-(γ,γ-dimethylallylamino) purine (2iP), kinetin, benzyladenine (BA), and *m*- topolin.

## Materials and Methods

The adenine-based cytokinins, trans-zeatin (tZ), 6-(γ,γ-dimethylallylamino) purine (2iP), kinetin, benzyladenine (BA), and *m*-topolin, (*Phyto*Technology Laboratories®, Overland Park, KS) used in these studies were >98% purity. Adenine-free base (*Phyto*Technology Laboratories®) was used as a model to predict behavior of these cytokinins in the analytical assays and was >99% purity.

**Figure 1.**
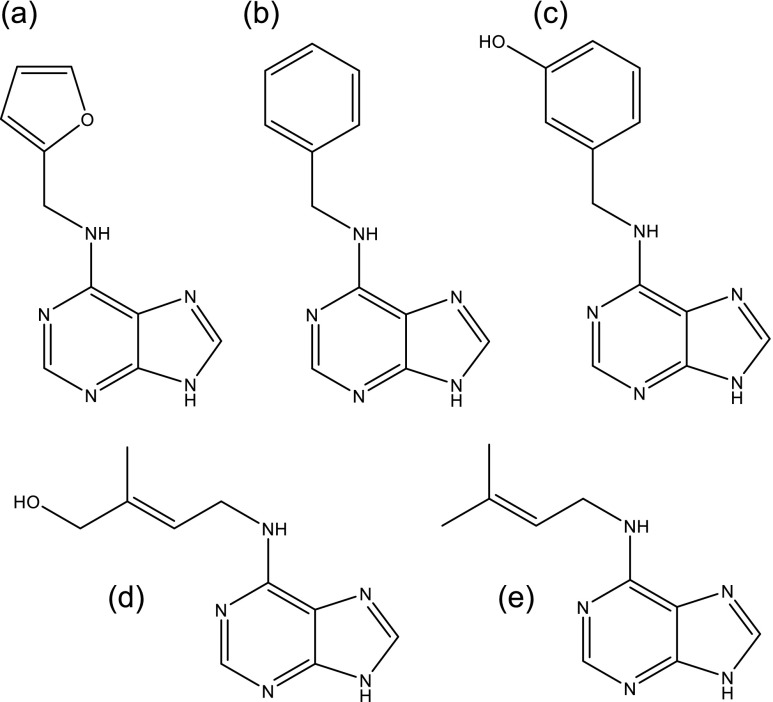
Structure of (*a*) kinetin, (*b*) BA, (*c*) *m*-topolin, (*d*) tZ, and (*e*) 2iP.

These cytokinins (Fig. 1) were dissolved in solutions containing a final concentration of 0.01–0.5 N KOH, depending on the cytokinin concentration, solubility, and ability to crystallize. The dissolution process occurred as follows to make a 1.0 mg mL^−1^ cytokinin solution at 0.05 N KOH; 1.0 mg of the powder was initially dissolved in 50 μL of 1.0 N KOH, vortexed until no powder was visible, and then brought to a final volume of 1 ml with 950 μL of deionized water (1–2 μS cm^−1^). Degradation studies used 50 mg mL^−1^ tZ in 0.5 N KOH and storage conditions of −20, 2–6, 25, 40, and 75°C. The other cytokinins were evaluated for stability over 3 mo at 1.0 mg mL^−1^ in 0.05 N KOH, except for tZ which was in 0.01 N KOH. The acid-based tZ study used 1.0 mg mL^−1^ tZ in 0.05 N HCl. Each sample used for stability analysis was stored in threaded amber glass vials capped with rubber septa and wrapped with teflon tape to minimize evaporation. Subsequent studies with tZ were performed at 85°C in 0.05 N KOH and 0.05 N HCl. Samples were stored at each temperature treatment for 90 d. Trials were performed in triplicate. One-way ANOVA was used to analyze the results obtained for each storage temperature treatment, and the *p* value is reported when the change is deemed statistically significant (*p* < 0.05).

Samples that were autoclaved were exposed to 121°C at 110 kPa for 30 min in a Sterilmatic STM-EL autoclave (Market Forge Industries, Everett, MA). The total time of exposure to temperatures above room temperature was approximately 1 h. Evaporation loss due to autoclaving was <10%. The loss for each sample was measured based on the total volume after cooling post-autoclave and was accounted for in the estimation of cytokinin concentration.

### ATR-FTIR (fourier transform infrared) spectroscopy

Spectra were obtained with a Spectrum Two FTIR spectrometer (Perkin Elmer, Waltham MA) with a LiTaO_3_ mini-infrared detector, a single-diamond universal ATR (attenuated-total reflectance) beam accessory, and a pressure arm equipped with a force gauge. The samples were blanked against their respective aqueous solution containing the appropriate concentration of KOH. A 5–20 μl aliquot of sample was placed on the ATR crystal and the spectra were recorded under 75 N of force. These spectra were recorded from an average of four scans, each with a resolution of 4 cm^−1^, at a scan speed of 0.2 cm s^−1^, and over the wavenumber range 4000–450 cm^−1^. Carbon dioxide band suppression was also used.

### High pressure liquid chromatography (HPLC)

HPLC was performed isocratically for each of the cytokinins with either 40% methanol (MeOH; for tZ and *m*-topolin) or 50% MeOH (for BA, kinetin, and 2iP) mobile phases at 0.85 mL min^−1^ on a DLC-20 Isocratic Liquid Chromatograph (Cole Parmer, Vernon Hills, IL). The MeOH used for these studies was ultrapure HPLC grade with ≥99.8% purity (Alfa Aesar, Ward Hill, MA). Mobile phases were either prepared in amber separate-from-use containers (with a low headspace volume above the liquid interface) or prepared fresh. Whether stored in containers to minimize evaporation or prepared fresh, the mobile phases were used for only 1 mo. The column used to resolve these peaks was a 25-cm-long (4.6-mm-diameter, 5-μm particles, 100-Å pore size) Kinetex® C18 column (Phenomenex, Torrence, CA) equipped with a security guard C18 cartridge. Chromatograms were monitored with UV absorbance at 270 nm. Peaks were isolated through fractionation based on the flow rate and volume of tubing exiting the UV-visible path length. The area under the curve (AUC) was determined to be linear with concentration across all five concentrations tested for each of the cytokinins (Fig. S1). This linearity allows for the concentration to be estimated by taking a proportion of the samples AUC to the initial AUC multiplied by the initial concentration.

### Repeated freeze-thaw cycling

To investigate the possibility of repeated freeze-thaw loss of tZ, two sets of samples of tZ at 1.0 mg mL^−1^ in 0.01 N KOH were set up in triplicate to be repeatedly frozen at−80°C and thawed at room temperature every 24 h. Also included were several triplicate control samples (control thaw) that were frozen once at day 0 with one of these samples being thawed and analyzed at every 24-h interval. The areas obtained from these freeze/thaw samples were normalized against the calibration curve area corresponding to 1.0 mg mL^−1^ tZ (*N* = 6) to provide a known outside standard area that was reproducible and not influenced by one single preparation. A single fresh preparation was assayed at 24-h intervals as a positive control.

### Mass spectrometry (MS)

For each cytokinin, electrospray ionization-mass spectrometry analysis ( ESI-MS) was performed on isolated HPLC peaks from degraded solutions as well as from pure cytokinin solutions. ESI-MS analysis was done on an LTQ linear Ion trap (Thermo Electron, Waltham, MA) with a nanospray source (Proxeon, Waltham, MA) with a picotip emitter (New Objective, Woburn, MA) via a 2 μL-sample loop (injection rotor) with a continuous infusion flowing at 4 μL min^−1^ (by syringe pump) of an aqueous 10% methanol, 50 mM acetic acid solution, collecting data in positive ion mode. Acquisitions (ESI-MS or ESI-MS/MS) were made using an optimized tune file generated with tZ infusion using similar conditions. Absolute detection of adenine and the cytokinins in the LTQ linear ion trap was assessed to verify that peak heights could be compared within spectra (cytokinin versus the adenine degradant peak). Each compound was diluted to a concentration of 100 pmol μL^−1^ and injected as described above. All of these related compounds produced very similar peak heights in the middle of the dynamic range of the ion trap in repetitive runs (data not shown). Therefore, similar conditions were used during the experiments.

## Results and Discussion

### Stability Studies with tZ in ATR-FTIR

**Figure 2.**
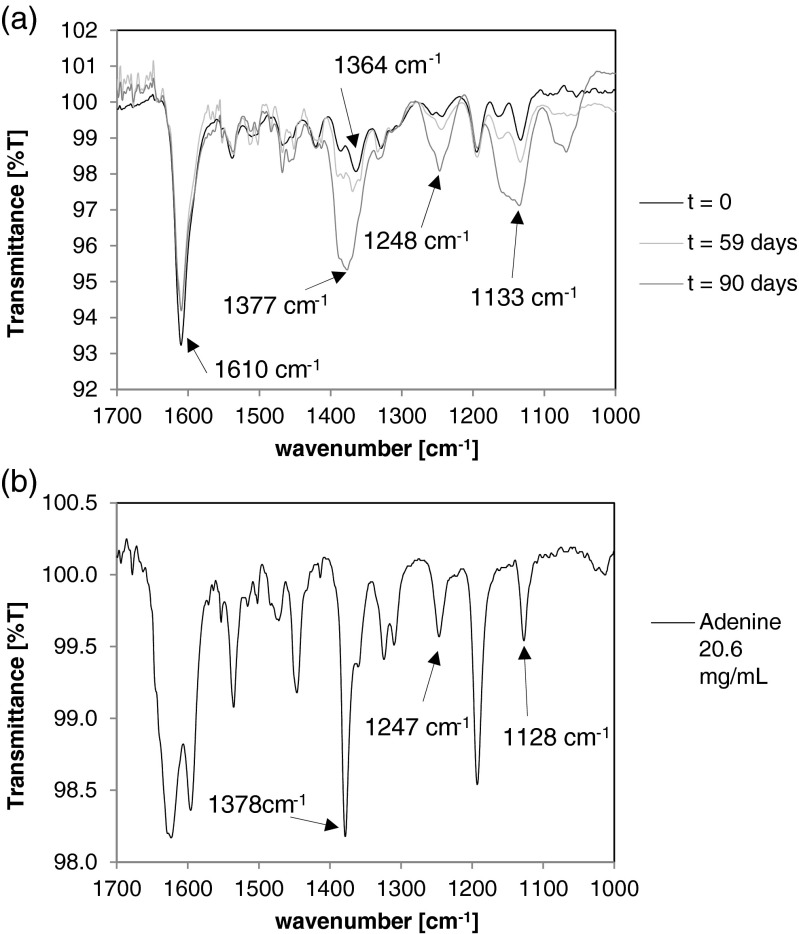
*a* FTIR spectra of an degradation study of tZ at 50 mg mL^−1^in 0.5 N KOH at 40°C. *b* Adenine in 0.3 N KOH at 20.6 mg mL^−1^.

Stability analysis was initially conducted using ATR-FTIR to monitor degradation since vibrational differences were expected to be found between the parent cytokinin and any degradants. The most intense peak in FTIR for adenine-based cytokinins in aqueous solution occurs at the frequency of 1610 cm^−1^ (Fig. 2*a* for tZ). This peak represents the signature of the secondary amines N-H movement on adenine. Also shown in Fig. 2*a* is change over time at 40°C: the 1610 cm^−1^ peak does slightly decrease with time, but not to the extent that the peak at 1364 cm^−1^ increases and shifts to 1377 cm^−1^ with time. Note the increase in peaks 1248 and 1133 cm^−1^ also in the degradation spectrum.

Adenine is a potential degradant from tZ after isoprenoid chain scission. Therefore adenine was dissolved in 0.3 N KOH at 20.6 mg mL^−1^ and the FTIR spectrum was recorded (Fig. 2*b*). The presence of the 1377, 1248, and 1133 cm^−1^ peaks provide evidence for the possibility of adenine accumulation in the degradation mixture since pure adenine also absorbs at these frequencies (Fig. 2*b*).

### HPLC characterization of parent tZ and degradants in aqueous solution

**Figure 3.**
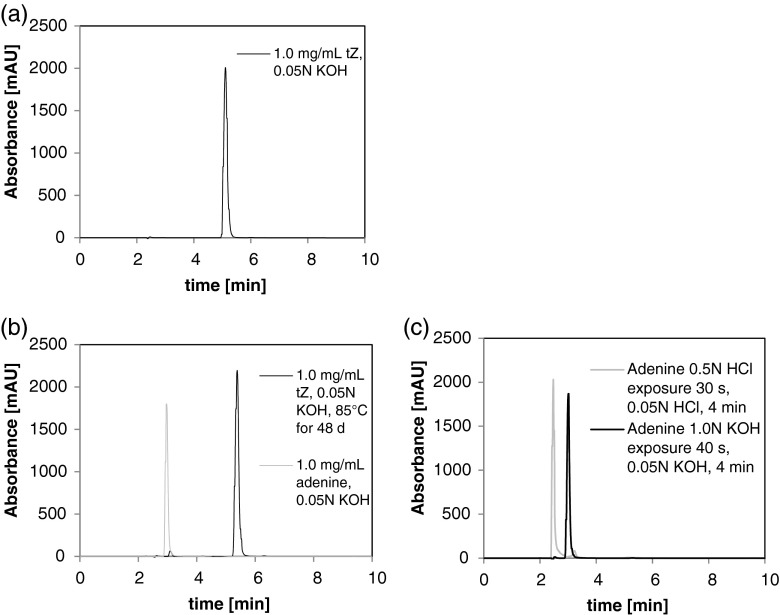
HPLC chromatogram of (*a*) standard tZ (1.0 mg mL^−1^, 0.05 N KOH), (*b*) overlap of adenine (near 3.0 min) with the 2.5 min degradant peak from tZ incubated at 85°C for 48 days, (*c*) adenine retention time shifting after exposure to 0.05 N HCl for 4 min relative to base exposure (0.05 N KOH) over the same time period.

HPLC was employed to quantify the degradation of these compounds because the limit of detection in FTIR-ATR was too high (15 mg mL^−1^). The typical HPLC chromatogram of pure tZ has a single peak eluting near 5 min (Fig. 3*a*). The only degradant that was seen was a small single peak eluting near 2–2.5 min as shown in Fig. 3*b*. In this instance, the degradant was obtained by incubating tZ in 0.05 N KOH at 85°C for 48 d. The small solvent peak in these chromatograms had retention times (*t*_r_ = 2 min) close to that of the degradant. Note the overlap between pure adenine dissolved in 0.05 N KOH at 1.0 mg mL^−1^ eluted near 3.0 min, with the degradant peak near the 2.5 min (Fig. 3*b*). It was difficult to obtain significant amounts of degradant with tZ in alkaline conditions at 1.0 mg mL^−1^ in 0.05 N KOH in degradation studies (Fig. 3*b*). Even samples incubated at 85°C did not show appreciable degradation after 1 mo relative to degradation with 0.5 N KOH exposures at 40°C for 90 d (data not shown).

Studies to examine the impact of acid hydrolysis on the adenine ring using preparations of pure adenine at room temperature in 0.05 N HCl suggested that degradation occurred in less than 3–4 min, approximately the time necessary to dissolve the powder and inject it onto the column for separation (Fig. 3*c*). The degraded adenine had an elution peak at 2.5 min, which would correspond to an elution volume of 2.1 mL, and was stable since the absorbance at 270 nm in HPLC was significant. The result of adenine degrading on such a short time span in a relatively low concentration of acid at room temperature supports the results seen by Lönnberg and Lehikoinen (1982).

### Mass spectrometry identification of tZ and degradants

**Figure 4.**
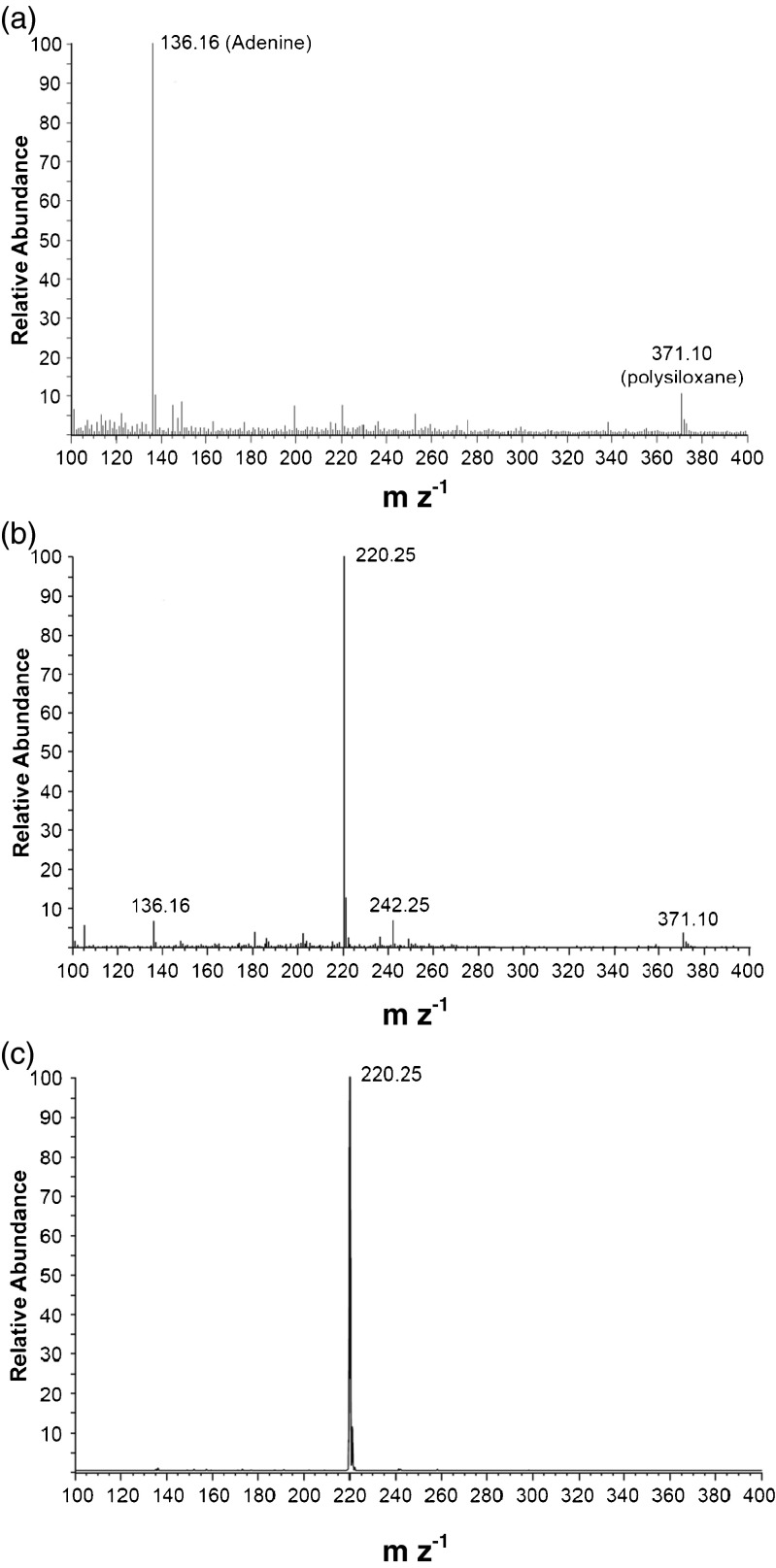
ESI-MS of (*a*) 1.8–3.0 min HPLC elution in 40% MeOH (136.16 m z-1 corresponds to adenine M + 1) from 50 μg of tZ in 0.5 N KOH, (*b*) 4.0–6.0 min HPLC elution in 40% MeOH (220.25 m z-1 corresponds to tz M + 1) from 50 μg of tZ in 0.5 N KOH, and (*c*) 5.2–5.5 min HPLC elution in 40% MeOH (220.25 m z-1 corresponds to tZ M + 1) from 2.5 μg of tZ in 0.05 N KOH.

Mass spectrometry was used to validate the peak assignments of these degradants obtained by HPLC profiles, and to further characterize these degradants. Following a study of tZ at 50 mg mL^−1^ in 0.5 N KOH incubated at 75°C for 34 d, 50 μg of tZ (based on the starting concentration) was injected onto the column and fractionated for mass spectrometry analysis. ESI-MS analysis in positive (+) ion mode of the isolated peak that eluted at 1.8–3.0 min (Fig. 3*b* at 2.5 min) revealed adenine as the primary degradation product (peak at 136.16 m z^−1^; Fig. 4*a*), with no tZ contamination. Polysiloxane (371.1 m z^−1^) is a common background ion generated in the loading of the sample.

ESI-MS analysis in positive (+) ion mode of the isolated tZ peak that eluted from 4.0–6.0 min revealed some residual adenine (peak at 136.1 m z^−1^) (Fig. 4*b*) with a sodium adduct of tZ (peak at 242.1 m z^−1^). However, the majority of the signal corresponded to tZ (peak at 220.2 m z^−1^). A substantial improvement in the resolution of tZ from adenine could be obtained by lowering both the concentration of the solution and volume injected (data not shown). To further test stability, a typical stock solution of 1.0 mg mL^−1^of tZ in 0.05 N KOH was stored at 85°C for 48 d and subjected to ESI-MS. A peak eluting at 5.2–5.5 min was observed to contain tZ at near-purity (peak at 220.25 m z^−1^, Fig. 4*c*).

To examine the impact of acid dissolution on HPLC peak composition, tZ was fractionated between 4.7–5.6 min of elution from a 1.0 mg mL^−1^ tZ in 0.05 N HCl solution that had been stored at room temperature for 56 d. ESI-MS of the isolated fraction showed results that were very similar to Fig. 4*c* (data not shown). Therefore for either alkaline or acidic dissolution, the peak eluting between 4.6–5.6 min (combining the acid and base overlap of elution) contained only residual adenine degradant.

Since only residual adenine was found in peaks fractionated between 4.6–5.6 min, peaks eluting in this retention time range from pure aqueous solutions of tZ were used to quantify the amount in solution.

### Mass spectrometry of parent cytokinin compounds

Chromatographic peaks were isolated for each of the other four cytokinins in the same manner as for tZ. Example mass spectra for each of these cytokinins are shown in supplementary Fig. S2. The apparent near-purity of the fractions obtained provided confidence that the chromatographic peak areas could be used to estimate the degree of degradation. Since the parent cytokinin peak area was also found to behave linearly with concentration, the ratio of the sample cytokinin peak area at and after time zero was the basis for cytokinin stability quantification.

### HPLC quantification of the stability of tZ in aqueous solution

**Figure 5.**
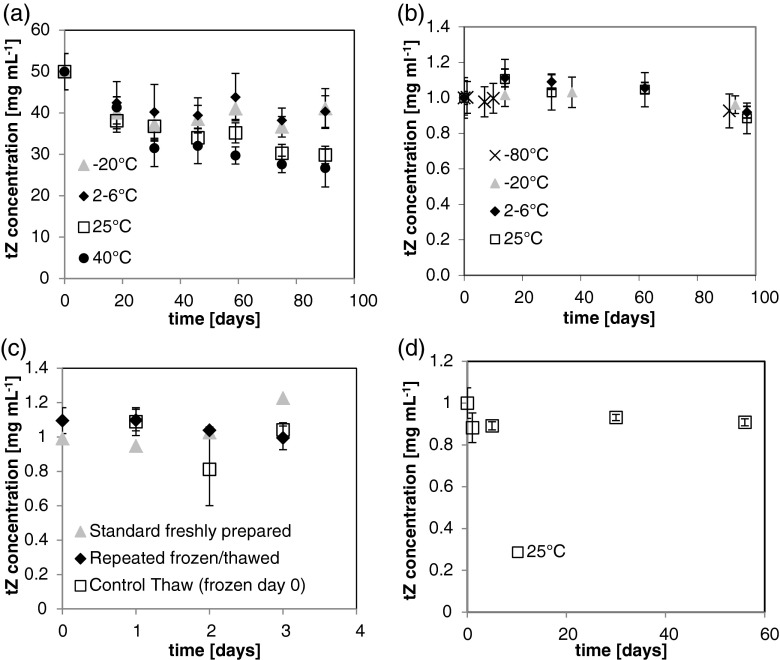
Stability profiles of trans-zeatin at (*a*) 50 mg ml^−1^ in 0.5 N KOH, (*b*) 1.0 mg ml^−1^ in 0.01 N KOH, (*c*) Freeze-thaw influence on stability of tZ at 1.0 mg ml^−1^ in 0.01 N KOH, and (*d*) 1.0 mg ml^−1^ in 0.05 N HCl. *Error bars* are plus and minus one standard deviation.

The peak area analysis for tZ at 50 mg mL^−1^ in 0.5 N KOH depicts the loss of mass with time at −20, 2–6, 25, and 40°C (Fig. 5*a*). There are two key trends shown in Fig. 5*a*. First, for each storage temperature treatment, tZ decayed to approximately 80% of initial concentration (40 mg mL^−1^) after 18 d. Second, tZ is stable at−20°C: it retained 80% (40 mg mL^−1^) of initial concentration through 90 d. Every storage temperature examined over time, except for the 2–6°C with 50 mg mL^−1^ tZ, resulted in highly statistically significant changes in tZ concentration (*p* < 0.001, 2–6°C *p* = 0.0504). Solutions of tZ at 50 mg mL^−1^ in 0.5 N KOH lost peak area and thus degraded faster than stock solutions of 1.0 mg mL^−1^ tZ and 0.01 N KOH (Fig. 5*b*). Though storage at 2–6°C of 1.0 mg mL^−1^ tZ in 0.01 N KOH caused a statistically significant concentration change (*p* = 0.03), storage at other temperatures studied over time did not result in any statistically significant change after 90 d. This is in stark contrast to the results obtained with 50 mg mL^−1^ tZ in 0.5 N KOH, and clearly the tZ solution is most stable when prepared at 1.0 mg mL^−1^ in 0.01 N KOH and stored at lower temperatures.

Since crystallization of BA and kinetin was seen, this required an investigation to rule out any possibility of freeze-thaw loss not able to be seen with the naked eye. Crystallization required the experiment to be halted because the concentration reported was from the dissolved cytokinin and degradation may be inferred when in fact the cytokinin may not be degraded, just not dissolved. Crystallization of BA and kinetin was sporadic and occurred following autoclaving a solution and storing it at 2–6°C before it had cooled to 25°C, or when samples were cooled to 25°C after they were heated significantly above ambient temperature (e.g., 75°C). This suggests the crystallization is a function of cooling rate, and nucleation site availability in the storage containers.

Neither the repeatedly frozen and thawed sample over three freeze-thaws, nor the control thaw showed any statistically significant concentration change over 3 d (Fig. 5*c*). From a kinetic point-of-view, the lowest temperature should provide the most stable storage. Therefore, in the absence of any freeze-thaw loss effects, tZ should be most stable at −80°C at 1.0 mg mL^−1^ in 0.01 N KOH.

Even though adenine immediately degraded in 0.05 N HCl during the course of sample preparation (3–4 min; Fig. 3*c*), tZ was stable in 0.05 N HCl for 56 d at 25°C (Fig. 5*d*). Nearly all of the tZ had degraded after storage in 0.05 N HCl at 85°C for 21 d (Data not shown), whereas multiple attempts to degrade tZ in 0.05 N KOH at 85°C for greater than 30 d left the molecule mostly intact (Fig. 3*b*).

### HPLC quantification of the stability of the adenine-based cytokinins, BA, kinetin, 2iP, and m-topolin

**Figure 6.**
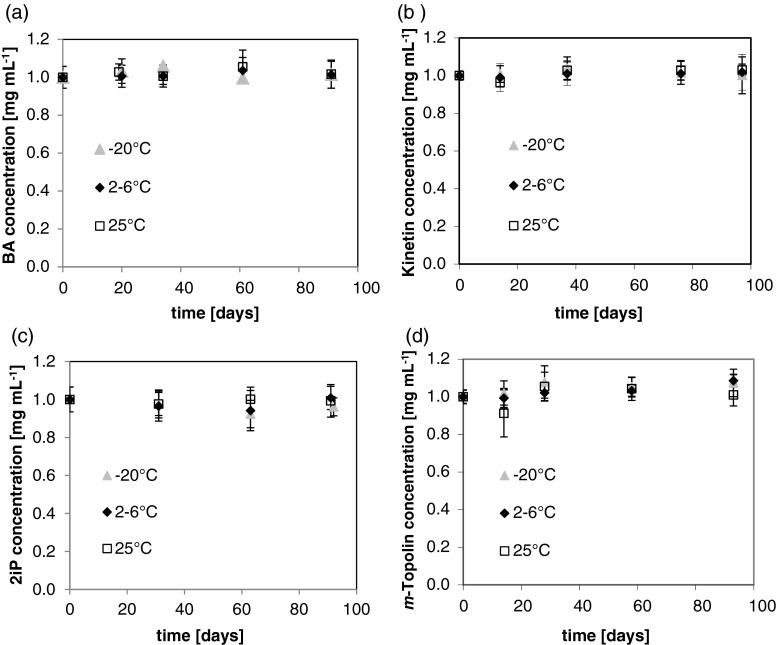
Stability profiles of (*a*) BA at 1.0 mg mL^−1^ in 0.05 N KOH, (*b*) kinetin at 1.0 mg mL^−1^ in 0.05 N KOH, (*c*) 2iP at 1.0 mg mL^−1^ in 0.05 N KOH, and (*d*) *m*-topolin at 1.0 mg mL^−1^ in 0.05 N KOH. None of the temperatures treatments resulted in statistically significant (*p* > 0.05) concentration change.

Crystallization during storage was less apparent with BA and kinetin when 0.05 N KOH was used for 1.0 mg mL^−1^ even if the cytokinin was initially soluble at 0.01 N KOH. Consequently, because of the stability of tZ in alkaline solution at 85°C, and because of crystallization issues with BA and kinetin in initial studies, 0.05 N KOH was used to prepare solutions of BA, kinetin, 2iP, and *m*-topolin (1.0 mg mL^−1^). Their stability was investigated over 90 d at storage temperatures of −20, 2–6, and 25°C. These are the most commonly used storage temperatures in plant tissue culture laboratories. No statistically significant (*p* > 0.05) concentration change was seen over all temperatures for the cytokinins BA, kinetin, 2iP, and *m*-topolin (Fig. 6). It is unknown why, among all of the cytokinins examined, tZ exhibited less stability with concentration changes being statistically significant for the 2–6°C at 1.0 mg mL^−1^ and 0.01 N KOH (Fig. 5*b*). The statistical significance of this change is not as much as was seen for the tZ degradation in 0.5 N KOH (Fig. 5*a*). Table 1 shows the times and temperatures for each of these cytokinins retaining greater than 90% of their initial concentration in alkaline solution.

**Table 1.** Summary of adenine-based cytokinin stability in alkaline solution

Cytokinin	KOH conc. [N]	Stabile (≥90%) [Y/N], Time [days]		
		−20°C	2–6°C	25°C
BA	0.05	Y, 91 days	Y, 91 days	Y, 91 days
Kinetin	0.05	Y, 98 days	Y, 98 days	Y, 98 days
2iP	0.05	Y, 92 days	Y, 91 days	Y, 91 days
tZ	0.01	Y, 93 days	Y, 97 days	Y, 62 days
	0.50	N, 18 days	N, 18 days	N, 18 days
*m*-topolin	0.05	Y, 94 days	Y, 94 days	Y, 94 days

### Physical stability of adenine-based cytokinins following one autoclave cycle and with MS salts

**Figure 7.**
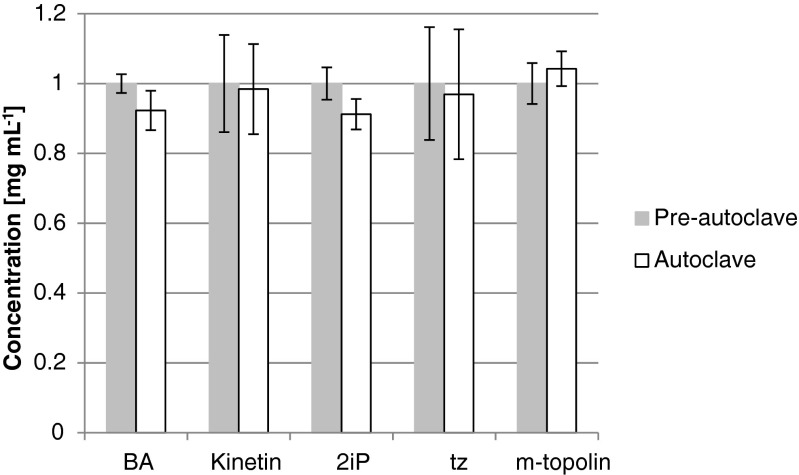
Autoclave stability of each of the cytokinins following one autoclave cycle (121°C, 110 kPa for 30 min).

Stability as affected by high temperatures and pressures, such as those within an autoclave cycle, is also of interest. Solutions of each cytokinin were prepared (1.0 mg mL^−1^, 0.05 N KOH in 20 mL of deionized water) and analyzed via HPLC prior to autoclaving. Post-autoclaving HPLC analysis did not show any peaks representative of degradation (Data not shown), nor were differences in concentrations detected relative to prior to autoclaving (Fig. 7). Since tZ was shown as the most unstable of these five cytokinins, its stability was examined in MS-basal salts after one autoclave cycle. There were negligible amounts of degradation of tZ when dissolved at 10 mg L^−1^ in standard MS-basal salts.

The data above showed that adenine-based cytokinins dissolved in relatively low base concentrations (≤0.05 N KOH) are stable in aqueous solutions for 90 d when stored at−20, 2–6, or 25°C. The amount of adenine recovered, based on approximate HPLC peak area relative to the parent tZ peak area, did not correlate with the amount of degraded tZ, but this was the only degradant that was identified with ATR-FTIR, HPLC elution volume, and ESI-MS. Based on the loss of peak area and thus absorbance at 270 nm for tZ at 0.5 N KOH, it is suggested that an adenine degradation mechanism resulted in the loss of 20–50% of the tZ over 90 d. This result is important because it is not uncommon to see plant tissue culture protocols instructing the dissolution of adenine-based cytokinins in 1.0 N KOH when as much as 20% by mass of the tZ can be lost over 18 d in 0.5 N KOH.

In 0.05 N HCl, tZ was stable for 56 d at 25°C, yet when pure adenine was dissolved at the same concentration of HCl at room temperature it degraded in 3–4 min (the time necessary to prepare the sample preparation and inject it into the HPLC column). Trans-zeatin did degrade in the same acid concentration at 85°C in 21 d, whereas very little breakdown product was evident from tZ in 0.05 N KOH at 85°C for greater than 30 d.

Manufacturers have traditionally considered the natural cytokinins tZ and 2iP to be heat-labile and specify storage temperatures of −20 and 2–6°C, respectively for these dry chemicals often without knowing their stability profiles. Yet plants synthesize natural cytokinins and other growth regulators through late stages of development and sometimes under harsh growing conditions (e.g., 40°C field conditions for agriculturally important plants like maize and wheat). While these compounds certainly can display heat-lability (especially when dissolved in 0.5 N KOH or 0.05 N HCl), powders stored in dry conditions with low-moisture content are more likely to be stable to the aqueous degradation mechanisms.

Adenine is relatively stable in aqueous solution. The results of autoclaving each of cytokinins showed no statistically significant concentration changes, which is consistent with adenine remaining intact after being exposed to 100°C for 1 h in 0.1 N–0.3 N NaOH (Jones *et al.*^1966^). Despite previous experience of autoclaving BA for tissue culture, there are only a few references to the physical stability of plant growth regulators in the literature. Perhaps more paradoxically is the noteworthiness of those citations. The first cytokinin discovered, kinetin, was found to become active after autoclaving from herring sperm (Miller *et al.*^1955^). Miller also tested exogenously applied zeatin riboside extracted from crown galls in a soybean-bioassay and found no difference in the results obtained whether sterilization was by autoclaving or filtration (Miller 1974), but did not attempt to quantitate or specifically measure zeatin riboside degradation. Though the ribosyl linkage to adenine may not have held up during autoclaving (Miller 1974), the tZ likely did, which is the basis for its cytokinin activity in the soybean assay. Though there is evidence for adenine-based cytokinin stability in a range of aqueous solution conditions and through one autoclave cycle in a moderately alkaline solution (0.05 N KOH), issues with crystallization, and potential adenine degradation under acidic conditions during extreme heating, should provide the user with caution in properly administering these compounds for plant tissue culture. Finally, when transferring tissue culture protocols, the exact powder dissolution method along with storage time and temperature should be considered.

## Electronic supplementary material

Fig S1 (DOCX 30 kb)

Fig S2 (DOCX 237 kb)

## References

[CR1] Arthur GD, Stirk WA, Novk O, Hekera P, van Standen J (2007). Occurrence of nutrients and plant hormones (cytokinins and IAA) in the water fern *Salvinia molesta* during growth and composting. Environ Exper Bot.

[CR2] Auer CA (2002). Discoveries and dilemmas concerning cytokinin metabolism. J Plant Growth Regul.

[CR3] Bairu MW, Kane ME (2011). Physiological and developmental problems encountered by *in vitro* cultured plants. Plant Growth Regul.

[CR4] Bairu MW, Novák O, Doležal K, Van Staden J (2011). Changes in endogenous cytokinin profiles in micropropagated *Harpagophytum procumbens* in relation to shoot-tip necrosis and cytokinin treatment. Plant Growth Regul.

[CR5] Besnard F, Refahi Y, Morin V, Marteaux B, Brunoud G, Chambrier P, Rozier F, Mirabet V, Legrand J, Lainé S, Thévenon E, Farcot E, Cellier C, Das P, Bishopp R, Parcy F, Helariutta Y, Boudaoud A, Godin C, Traas J, Guédon Y, Vernoux T (2014). Cytokinin signaling inhibitory fields provide robustness to phyllotaxis. Nature.

[CR6] Bollmark M, Elisasson L (1986). Effects of exogenous cytokinins on root formation in pea cuttings. Physiol Plantarum.

[CR7] Brandstatter I, Kieber JJ (1998). Two genes with similarity to bacterial response regulators are rapidly and specifically induced by cytokinin in Arabidopsis. Plant Cell.

[CR8] Frébort I, Kowalska M, Hluska T, Frébortová J, Galuszka P (2011). Evolution of cytokinin biosynthesis and degradation. J Exp Bot.

[CR9] Gautheret RJ (1983). Plant tissue culture: a history. Bot Mag Tokyo.

[CR10] Hwang I, Sheen J (2001). Two-component circuitry in Arabidopsis cytokinin signal transduction. Nature.

[CR11] Iliev I, Gajdošová A, Liviaková G, Jain SM, Davey MR, Anthony P (2010). Plant micropropagation. Plant cell culture: essential methods.

[CR12] Jones AS, Mian AM, Walker RT (1966) The action of alkali on some purines and their derivatives. J Chem Soc C 692–695

[CR13] Kaeppler SM, Phillips RL (1993). DNA methylation and tissue culture-induced variation in plants. In Vitro Cell Dev Biol - Plant.

[CR14] Kakimoto T (1996). CKI1, a histidine kinase homolog implicated in cytokinin signal transduction. Science.

[CR15] Larkin PJ, Scowcroft WR (1981). Somaclonal variation—a novel source of variability from cell cultures for plant improvement. Theor Appl Genet.

[CR16] Letham DS, Palni LMS (1983). The biosynthesis and metabolism of cytokinins. Annu Rev Plant Physiol Plant Mol Biol.

[CR17] Lönnberg H, Lehikoinen P (1982). Mechanisms for the solvolytic decompositions of nucleoside analogues. X. Acidic hydrolysis of 6-substituted 9-(β-D-ribofuranosyl) purines. Nuc Acids Res.

[CR18] Miller CO (1974). Ribosyl-*trans*-zeatin, a major cytokinin produced by crown gall tumor tissue. Proc Natl Acad Sci U S A.

[CR19] Miller CO, Skoog F, Von Saltza MHV, Strong FM (1955). Kinetin, a cell division factor from deoxyribonucleic acid. J Am Chem Soc.

[CR20] Mok MC, Martin RC, Dobrev PI, Vanková R, Ho PS, Yonekura-Sakakibara K, Sakakibara H, Mok DWS (2005). Topolins and hydroxylated thidiazuron derivatives are substrates of cytokinin *O*-glucosyltransferase with position specificity related to receptor recognition. Plant Physiol.

[CR21] Mok DWS, Mok MC (2001). Cytokinin metabolism and action. Annu Rev Plant Physiol Plant Mol Biol.

[CR22] Murashige T (1974). Plant propagation through tissue culture. Annu Rev Plant Physiol Plant Mol Biol.

[CR23] Panaia M, Bunn E, McComb J (2011). Primary and repetitive secondary somatic embryogenesis of *Lepidosperma drummondii* (Cyperaceae) and *Bloskion tetraphyllum* (Restionaceae) for land restoration and horticulture. In Vitro Cell Dev Biol - Plant.

[CR24] Sakakibara H (2006). Cytokinins: activity, biosynthesis, and translocation. Annu Rev Plant Physiol Plant Mol Biol.

[CR25] Shani E, Yanai O, Ori N (2006). The role of hormones in shoot apical meristem function. Curr Opin Plant Biol.

[CR26] Shukla A, Sawhney VK (1992). Cytokinins in a genic male sterile line of *Brassica napus*. Physiol Plantarum.

[CR27] Skoog F, Armstrong DJ (1970). Cytokinins. Annu Rev Plant Physiol Plant Mol Biol.

[CR28] Skoog F, Miller CO (1957). Chemical regulation of growth and organ formation in plant tissues cultured *in vitro*. Symposia of the Society for Experimental Biology Number XI. The biological action of growth substances.

[CR29] Smulders MJM, De Klerk GJ (2011). Epigenetics in plant tissue culture. Plant Growth Regul.

[CR30] Takei K, Sakakibara H, Sugiyama T (2001). Identification of genes encoding adenylate isopentyltransferase, a cytokinin biosynthesis enzyme, in *Arabidopsis thaliana*. J Biol Chem.

[CR31] Takei K, Yamaya T, Sakakibara H (2004). Arabidopsis CYP735A1 and CYP735A2 encode cytokinin hydroxylases that catalyze the biosynthesis of trans-zeatin. J Biol Chem.

[CR32] Tanurdzic M, Vaughn MW, Jiang H, Lee TJ, Slotkin RK, Sosinski B, Thompson WF, Doerge R, Martienssen W (2008). Epigenomic consequences of immortalized plant cell suspension culture. PLoS Biol.

[CR33] Thorpe TA, Loyola-Vargas VM, Vázquez-Flota F (2006). History of plant tissue culture. Methods in molecular biology, plant cell culture protocols, v 318.

[CR34] Van Staden J, Drewes SE, Hutton MJ (1982). Biological activity of 6-(2,3,4-trihydroxy-3-methylbutylamino) purine, an oxidation product of zeatin. Physiol Plantarum.

[CR35] Van Staden J, Forsyth C (1985). Peroxidase and zeatin stability. J Plant Physiol.

[CR36] Werner T, Motyka V, Laucou V, Smets R, Van Onckelen H, Schmülling T (2003). Cytokinin-deficient transgenic Arabidopsis plants show multiple developmental alterations indicating opposite functions of cytokinins in the regulation of shoot and root meristem activity. Plant Cell.

[CR37] Wickson M, Thimann KV (1958). The antagonism of auxin and kinetin in apical dominance. Physiol Plantarum.

