# Supplementary material for: Stability of adenine-based cytokinins in aqueous solution
Source: In Vitro Cell Dev Biol Plant. 2016 Feb 4;52:1–9. doi: 10.1007/s11627-015-9734-5 (PMC4759223; doi:10.1007/s11627-015-9734-5)
Supplement: Supplementary file 1 — (DOCX 30 kb) [file 11627_2015_9734_MOESM1_ESM.docx]

(b)

(a)

(e)

(d)

(c)

Figure S1. Calibration curves of parent cytokinin peak area relative to parent cytokinin concentration prior to dilution for the sample preparation (i.e. actual concentration injected is 4X less); (a) kinetin, (b) benzyladenine, (c) *m*-topolin, (d) *trans*-zeatin, and (e) 2iP.
