# Supplementary material for: Stability of adenine-based cytokinins in aqueous solution
Source: In Vitro Cell Dev Biol Plant. 2016 Feb 4;52:1–9. doi: 10.1007/s11627-015-9734-5 (PMC4759223; doi:10.1007/s11627-015-9734-5)
Supplement: Supplementary file 2 — (DOCX 237 kb) [file 11627_2015_9734_MOESM2_ESM.docx]

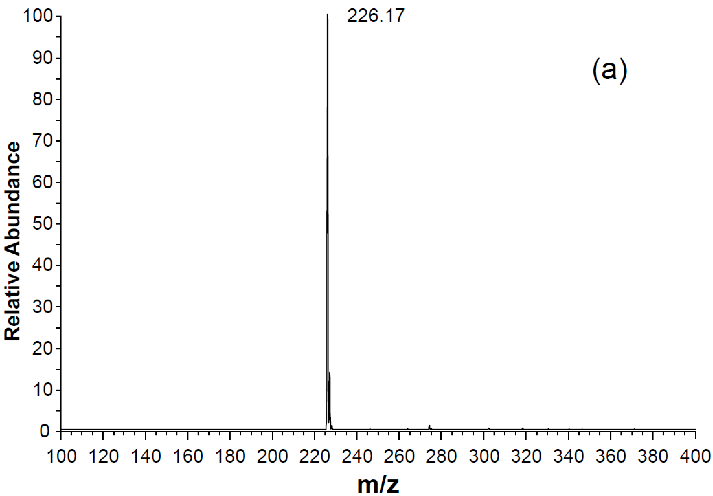

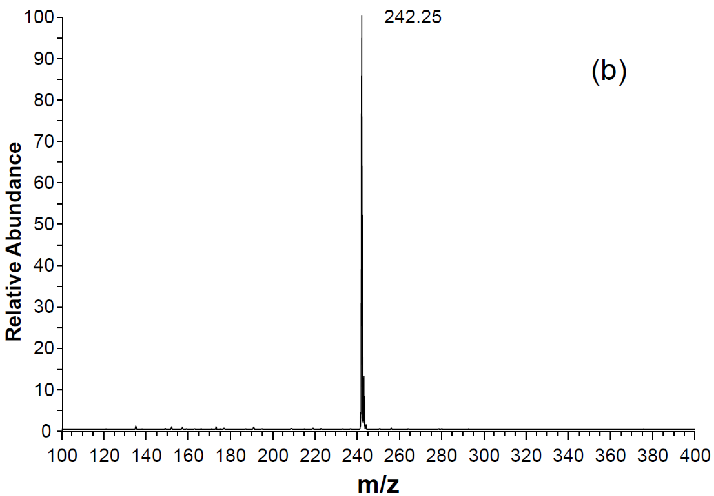


**m z^-1^**

**m z^-1^**


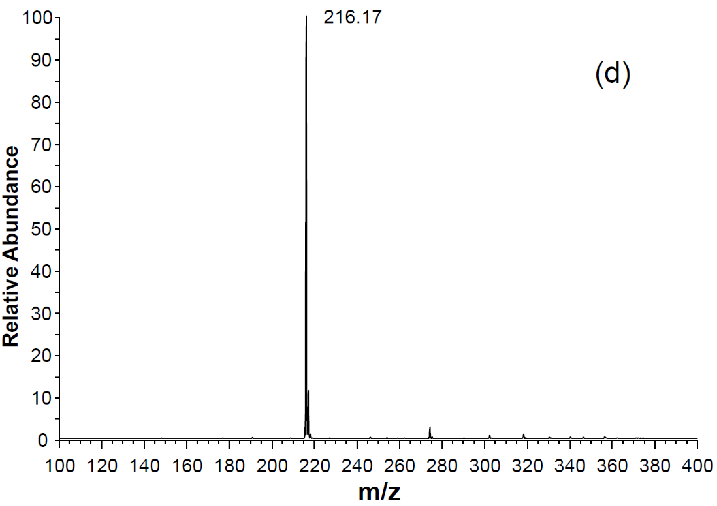

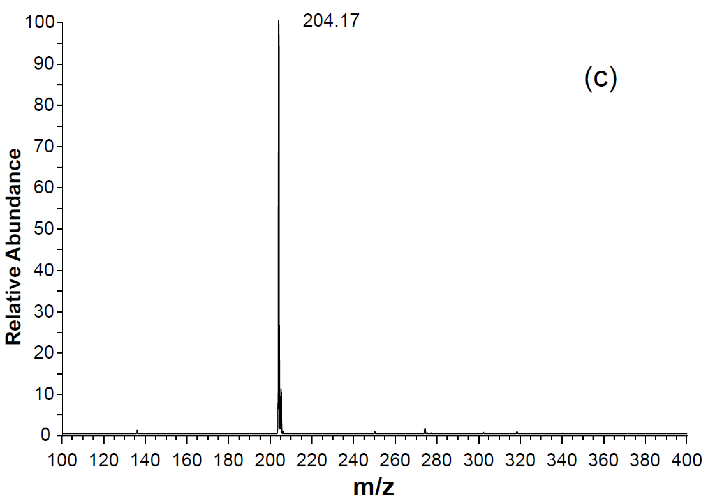


**m z^-1^**

**m z^-1^**

Figure S2. ESI of (a) 10.1-10.6 min BA in HPLC elution in 50% MeOH (226.17 m z^-1^ corresponds to BA M+1), (b) 8.0-8.3 min *m*-topolin in HPLC elution in 40% MeOH (242.25 m z^-1^  corresponds to *m*-topolin M+1), (c) 11.7-12.1 min 2iP in HPLC elution in 50% MeOH (204.17 m z^-1^  corresponds to 2iP M+1) (d) 5.5-5.8 min kinetin in HPLC elution in 50% MeOH (216.17 m z^-1^  corresponds to kinetin M+1)
